# Introduction of an Alcohol-Related Electronic Screening and Brief Intervention (eSBI) Program to Reduce Hazardous Alcohol Consumption in Namibia’s Antiretroviral Treatment (ART) Program

**DOI:** 10.1007/s10461-019-02648-9

**Published:** 2019-08-23

**Authors:** A. M. Tang, N. Hamunime, R. A. Adams, G. Kanyinga, C. Fischer-Walker, S. Agolory, D. Prybylski, N. Mutenda, S. Sughrue, D. D. Walker, T. Rennie, M. Zahralban-Steele, A. Kerrigan, S. Y. Hong

**Affiliations:** 1 School of Medicine, Tufts University, Boston, MA USA; 2 Government of Namibia, Ministry of Health and Social Services, Windhoek, Namibia; 3 U.S. Centers for Disease Control & Prevention, Atlanta, GA USA; 4 School of Social Work, University of Washington, Seattle, WA USA; 5 School of Pharmacy, University of Namibia, Windhoek, Namibia; 6 Department of Public Health and Community Medicine, Tufts University School of Medicine, 136 Harrison Avenue, MV248, Boston, MA 02111 USA

**Keywords:** Alcohol, HIV, Screening and brief intervention, Namibia

## Abstract

Alcohol is the most widely abused substance in Namibia and is associated with poor adherence and retention in care among people on antiretroviral therapy (ART). Electronic screening and brief interventions (eSBI) are effective in reducing alcohol consumption in various contexts. We used a mixed methods approach to develop, implement, and evaluate the introduction of an eSBI in two ART clinics in Namibia. Of the 787 participants, 45% reported some alcohol use in the past 12 months and 25% reported hazardous drinking levels. Hazardous drinkers were more likely to be male, separated/widowed/divorced, have a monthly household income > $1000 NAD, and report less than excellent ART adherence. Based on qualitative feedback from participants and providers, ART patients using the eSBI for the first time found it to be a positive and beneficial experience. However, we identified several programmatic considerations that could improve the experience and yield in future implementation studies.

## Resumen

El alcohol es la sustancia que más se abusa en Namibia y la cual está asociada con baja adherencia a la terapia antiretroviral (TAR) y retención pobre en los cuidados de salud entre las personas que están recibiendo TAR. La prueba de detección electrónica y las intervenciones breves (eSBI) son efectivas para reducir el consumo de alcohol en diversos contextos. Se utilizó un diseño de métodos mixtos para desarrollar, implementar y evaluar la introducción de un eSBI en dos clínicas de TAR en Namibia. De los 787 participantes, el 45% reportó algún consumo de alcohol en los últimos 12 meses y el 25% reportó niveles peligrosos de consumo de alcohol. Aquéllos que consumieron alcohol en cantidades peligrosas tenían la tendencia a ser hombres, estar separados/viudos/divorciados, tener un ingreso familiar mensual > $ 1000 NAD y reportar adherencia al TAR menos que excelente. De acuerdo a los comentarios cualitativos de los participantes y los proveedores, los pacientes con TAR que utilizaron el eSBI por primera vez consideraron que la experiencia fue positiva y beneficiosa. Sin embargo, identificamos varias consideraciones programáticas que podrían mejorar la experiencia y el rendimiento en futuros estudios de implementación.

## Introduction

Namibia, a sub-Saharan country in southwest Africa, has been severely affected by the HIV epidemic. With approximately 217,000 people living with HIV (PLHIV), Namibia has one of the world’s highest HIV prevalence rates, estimated to be approximately 14% in the general adult population and up to 23.7% in the most heavily affected region in the north [1]. According to the World Health Organization (WHO), HIV/AIDS is the leading cause of death in Namibia [2].

Alcohol is the most widely abused substance in Namibia [3]. The 2014 WHO Global Status Report on Alcohol and Health reported the prevalence of alcohol use disorders and alcohol dependence in Namibia to be significantly higher compared to the overall African region (5.1% vs. 3.3% and 2.2% vs. 1.4%, respectively) [4]. In addition, Namibia’s alcohol per capita consumption and prevalence of heavy episodic (binge) drinking ranked as the third and sixth highest, respectively, out of 45 countries in the African region [4].

Heavy alcohol use among PLHIV is of major public health concern because it is associated with increased risk of HIV transmission through diminished personal control and increased likelihood of engaging in unprotected sexual activity [5–11]. Efforts to curb heavy alcohol consumption among PLHIV are critical to maintaining the HIV continuum of care. In a systematic review, Vagenas et al. identified 53 studies that examined the effects of alcohol use on various points along the HIV care continuum [12]. While there were various limitations among the individual studies and lack of consistency in measurement and definitions of alcohol use across studies, 77% of the studies reported that alcohol negatively impacted one or more stages of the HIV care continuum. More than half of the studies conducted in low- and middle-income countries found a negative association between alcohol use and viral suppression.

Alcohol interventions may have greater impact if targeted at patients attending antiretroviral treatment (ART) clinics, considering these individuals may be more inclined to change their negative health behaviors than PLHIV who are not engaged in care [11, 13]. Alcohol-related screening, brief intervention and referral to treatment (SBIRT) is an effective, evidence-based intervention that can be utilized by health care professionals and trained personnel [14, 15]. A structured set of questions is used to screen and identify individuals at risk for alcohol use problems. Screening is followed by a brief intervention and, if needed, referral to treatment. The brief intervention is generally a 5- to 15-min counseling session to reduce risky drinking using motivational interviewing techniques. In settings where referrals to treatment are limited, research suggests that, alcohol-related screening and brief interventions (SBIs) alone can effectively identify alcohol-related problems and decrease their severity [16].

Electronic (computerized) SBIs (eSBIs) use technology to deliver SBI content that is traditionally delivered face-to-face with a trained counselor. eSBIs are effective in decreasing alcohol consumption and have demonstrated the ability to resolve many of the barriers related to implementing counselor-driven SBIs, including limited resources, time, and capacity for specialized training [17]. Additionally, eSBIs have been shown to decrease costs associated with intervention delivery, enhance standardization, and decrease implementation-related issues when compared to face-to-face SBIs [16, 17]. eSBIs offer the ability to provide an individualized intervention, but with the added benefit of reducing the frequency of underreporting due to stigma [16]. Therefore, there is potential for eSBIs to provide a more standardized assessment of alcohol consumption, along with individualized goals and strategies for reducing risky drinking. In addition, the eSBI is an attractive intervention because it allows for streamlined implementation in busy ART clinics in resource-limited settings. We developed and piloted an alcohol-related eSBI specific to the Namibian context, with the overall objectives of: (1) screening for hazardous alcohol use and associated risk factors among PLHIV attending two ART clinics (in the North and Central regions), and (2) assessing the feasibility and acceptability of implementing this type of tool in ART sites in Namibia.

## Methods

### Study Design

This pilot project involved active collaboration with the Republic of Namibia’s Ministry of Health and Social Services (MoHSS) Directorate of Special Programmes (HIV/AIDS, TB, and malaria) and the U.S. Centers for Disease Control and Prevention (CDC) office in Namibia. We used a mixed methods approach to develop, implement, and evaluate the introduction of an alcohol-related eSBI in two ART sites in Namibia: Katutura Health Centre in the capital city of Windhoek, and Oshakati State Hospital in Oshakati, the regional capital of the Northern region of Oshana. The regions were chosen by the MoHSS to represent two geographic areas experiencing significant problems with both HIV and hazardous alcohol use. Within these regions, the two ART sites were selected based on their size and capacity to accommodate the eSBI intervention. A convenience sample of patients was recruited at each site to use the self-administered eSBI monthly for a period of 3 months during routinely scheduled clinic visits. After 3 months of implementation, we conducted focus group discussions (FGDs) with the ART providers and individual interviews with patients to evaluate their experiences with this tool. This study was reviewed according to the Centers for Disease Control and Prevention (CDC) human research protection procedures and determined to be research. This study was also reviewed and approved by the Health Sciences Institutional Review Board of Tufts University and the Biomedical Research Ethics Committee of the Republic of Namibia MoHSS.

### Content of the eSBI

Prior to programming the eSBI, qualitative data obtained from FGDs with both ART patients and providers were used to inform and adapt the development of a brief intervention script specific to the Namibian context. The original script, based on a computerized alcohol screening and intervention developed by Vaca et al. [18] was modified according to key terms and themes elicited from the FGDs revealing motivations for alcohol use among PLHIV in Namibia. Briefly, we made several types of changes: (1) We substituted several terms and phrases with those more commonly used in Namibia. For example, “taking ARV medications” was changed to “drinking ARV medications”; “small beer” was changed to “dumpie”; “traditional homebrew” was changed to “tombo”; “bar” was replaced with “shebeen”; and the term “tot” was used in place of “shot”; (2) The risk scale (“low, moderate and high risk”) was modified to “low, middle, and high danger” as the term “danger” was said to be better understood than the term “risk”; (3) We created and incorporated graphics of standard alcohol types and standard drink volumes using bottles and glasses that would be more widely recognized by Namibians; (4) We incorporated reasons and ideas for limiting alcohol use that were elicited from our FGDs; and (5) We provided participants with local resources that they could contact for additional help.

We then developed our own computerized version of the modified SBI script. The SBI script was translated and programmed in the three most commonly spoken languages in the two regions—English, Afrikaans, and Oshiwambo. Audio in the three languages was incorporated into the program so that participants could hear the questions read aloud to them through headphones.

#### Introduction

The eSBI began with a brief tutorial on the various functionalities of the computer program followed by a few demographic and health questions including gender, primary language, education, marital status, employment status, monthly household income, current ART status, and ART adherence in the past month. ART adherence was assessed using the following question, “For some people, it is difficult to always drink ARV medications as the doctor prescribes. Thinking back over the past month, please tell me how well you think you drank all of your ARV medications as prescribed?” Responses were recorded on a five-point Likert scale, “Excellent, Very Good, Good, Fair, or Poor”.

#### Alcohol Screening Tool

The screening tool used in our eSBI was the WHO’s Alcohol Use Disorders Identification Test (AUDIT) questionnaire [19]. The AUDIT is a 10-item questionnaire that assesses alcohol use during the previous 12 months and indicates the presence and severity of an alcohol problem or alcohol use disorder. Questions are grouped into three domains: (1) hazardous alcohol use (frequency of drinking, typical quantity of alcohol consumed, and frequency of heavy (binge) drinking; (2) alcohol dependency syndromes (impaired control over drinking, increased salience of drinking, and morning drinking); and (3) harmful alcohol use (guilt after drinking, blackouts, alcohol-related injuries, and others concerned about drinking). Each of the 10 questions is rated on a 3- or 5-point scale with total scores ranging from 0 to 40. In the original development of the AUDIT tool, a cutoff score of 8 was found to be a valid indicator of hazardous and harmful alcohol use in primary health care centers in six countries (Australia, Bulgaria, Kenya, Mexico, Norway, and the USA) [19, 20]. The cutoff score of 8 has also been validated against the Mini-International Neuropsychiatric Interview (MINI) in PLHIV in Zambia and South Africa [21, 22]. Since participants were engaging with the eSBI on a monthly basis for a period of 3 months, we modified the AUDIT questionnaire for the follow-up visits to capture alcohol use disorders over the previous 1-month period.

#### Brief Intervention

Participants who scored in the low risk category (AUDIT score 0–7) received positive feedback and skipped directly to the eSBI feedback questions on usability and acceptability. At-risk participants (defined as AUDIT score ≥ 8) went on to the brief intervention section of the program. This section began with a restating of the frequency of drinking that the participant self-reported, followed by normative feedback questions asking participants to indicate the number of Namibian men/women (out of 10) they believed: (1) drank any alcohol in the past year, and (2) drank 6 or more drinks at least one time in the past month. After each question, participants were provided with the true frequencies in Namibia based on a national survey [4]. Participants were then given information on reasons why people in Namibia drink and how drinking large amounts of alcohol can cause problems with their ability to manage their HIV medication, their overall health and their family and social lives. Following this, participants were assessed for their readiness to reduce their alcohol consumption using a single question: “How ready are you to limit the amount of alcohol you drink?” Possible responses were “Not ready”, “Somewhat ready”, or “Very ready”. Those who indicated they were “somewhat ready” or “very ready” were asked to select from a list of all the reasons why they wanted to limit the amount of alcohol they drank. Next, these participants were offered a list of targeted goals to commit to including: (1) limiting (reducing) the amount of alcohol consumed; (2) drinking no more than the recommended amounts (no more than 3 standard drinks in a day for men and 2 standard drinks in a day for women, and to abstain from drinking for at least 2 consecutive days per week); (3) stopping drinking altogether; or (4) not able to make a commitment at this time. These goal options were anchored on the Namibian government’s recommendations for healthy drinking (option 2). Option 1 was provided to reinforce any moderation the participant was willing to make and option 3 was provided to reinforce an abstinence goal. This goal strategy is consistent with the WHO’s recommendations for brief interventions for alcohol [23]. Finally, participants were presented with a list of strategies to help them limit the amount of alcohol they drink. Participants were able to select the strategies they wanted to learn more about.

Following the eSBI, all participants received a personalized paper report stating the risk category they fell into, the goal they committed to, and some general information on recommended drinking levels, standard drink amounts, and warning information such as not drinking during pregnancy or prior to driving or doing anything that requires concentration. Participants were also given a list of appropriate resources to seek further help based on their location and risk category.

#### eSBI Feedback Questions

Eight feedback questions on the usability and acceptability of the eSBI program were asked of all participants at the end of the program. Responses were on a 5-point Likert scale ranging from 1 (strongly agree) to 5 (strongly disagree). The statements addressed the ease of use, program length, helpfulness, and preference compared to speaking to a doctor or health care worker about their drinking (see Table 4 for full list of statements). In addition, the program recorded the amount of time (in seconds) it took each participant to complete the eSBI.

### Participant Enrollment

HIV patients attending their routinely scheduled clinic visit at the two selected ART sites were enrolled over a 1-month period. Eligibility criteria included age ≥ 18 years, on ART, and able to verbally communicate in English, Oshiwambo, or Afrikaans. Patients who were waiting in a queue to see the doctor, nurse, or pharmacist were approached by a study coordinator and told briefly about the study. Eligible patients who were interested in participating were removed from the queue with the assurance that they would be “fast-tracked” to the front of the queue after completing the study. Informed consent was administered to all participants prior to administration of the eSBI. Participants were informed that, depending on their level of alcohol use, the following may occur after going through a 10–15 min computer program on alcohol use: (a) the program will simply help them to reflect on their alcohol use; (b) the program would educate them on the harms of alcohol use and help them to consider reducing their alcohol use; and/or (c) the program would refer them for further treatment if requested or necessary. They were also told that they would receive a printed copy of their results. Monetary compensation was not given to these participants.

After 3 months of eSBI implementation, we conducted individual interviews with ten randomly selected patients from each site to gather more detailed information on their experience using the eSBI program. After the eSBI program had been operating for 3 months in the clinics, patients who had participated were identified and invited by phone to participate in an interview with the study coordinator. Those who were interested were scheduled to meet with the interviewer at the clinic where the study procedures were reviewed, and informed consent administered.

We also conducted FGDs with providers to obtain their perspectives on how well the program was integrated into clinic services. All providers who were present at the two ART sites were invited to participate in FGDs. We aimed to conduct one FGD at each site; however, at Katutura Health Center, only two out of six providers were available for the FGD on the day the interview took place. The FGD at Oshakati State Hospital included five providers (out of 10 total). All FGD participants were consented into the study at the beginning of each FGD session. All interview and FGD participants were compensated 50 Namibian dollars (equivalent to 3.50 USD) for their time.

### Data Collection

#### Quantitative Component

Participants navigated the eSBI on their own via a laptop computer, using headphones to hear the questions privately and a touch-screen to select their responses. Quantitative data were directly downloaded from the eSBI program and included information on demographics, ART use, AUDIT screening, eSBI feedback questions, and time spent navigating through the eSBI program. Given low uptake of the eSBI at the monthly follow-up visits (48% at the 1st follow-up visit, 14% at the 2nd follow-up visit, and 4% at the 3rd follow-up visit), this paper reports only on the quantitative data downloaded from the baseline visit. Potential reasons for the low uptake at follow-up visits are explained in more detail later in the paper.

#### Qualitative Component

Semi-structured interview guides were developed and used for the FGDs with ART providers and the individual interviews with patients. FGD questions included how well the providers thought the eSBI was incorporated into routine clinic functions (“How well do you think the eSBI was incorporated into the routine clinic functions? What worked well and what didn’t work well?”) and whether it was beneficial for the patients (“Do you think eSBI was beneficial for patients in decreasing alcohol consumption? Please explain”); their impressions of how the patients felt about the program (“How do you think the patients felt about going through the eSBI program? Did you hear any positive or negative feedback about the program?”); the logistics of how the eSBI could be incorporated into future clinical care at the clinic (“Now that the study is done, do you see eSBI as something that could be incorporated into the routine clinical care of patients? How can this happen? What are the potential barriers?”); and the importance and sustainability over the long-term (“Do you think eSBI is important to the long-term success of the ART program?”).

The individual interviews focused on patients’ experiences navigating through the program (e.g. “How was your experience using the alcohol program?”, “What was the most difficult part of using the alcohol program?”, “Was the alcohol program too long, too short, or just right?”); their understanding of the questions, graphics, and any alcohol advice given (“Did you understand the questions and the alcohol advice?”, “Were the translations good?”, “Did you enjoy the pictures/videos in the alcohol program? Were they helpful or distracting?”; whether the program motivated them to reduce their alcohol consumption (“Did the alcohol program affect your life in any way?”, “Did the alcohol advice help you to want to reduce your alcohol consumption? What did you do to reduce your alcohol consumption?”, “Did you use any of the alcohol advice given to you by the computer?”); challenges they faced in reducing their alcohol consumption (“What are the difficulties/challenges to reducing your alcohol consumption?”); participation in the program at the follow-up visits (“Did you want to participate in the alcohol program at your follow-up visits?”, “Was it more difficult or was it easier for you to participate in the alcohol program the second or third time?”; and suggestions on how to improve the program (“Is there anything you can suggest to make the alcohol program better?”).

FGDs at both clinics lasted approximately 1 h and were conducted in English by SYH with the support of a research assistant. The individual interviews with patients lasted about 15 min each and were conducted by local study coordinators. All FGDs and individual interviews were audio-recorded and later transcribed verbatim and translated into English by a research assistant.

### Data Analysis

#### Quantitative Component

Total AUDIT scores were calculated, and participants were categorized into low risk (0–7), medium risk (8–15), high risk (16–19), and dependent (20–40) based on their baseline AUDIT score [19]. We also categorized participants into risk categories based on their answers to specific AUDIT questions: (1) Non-drinker, no risk; (2) Non-drinker, with past or recent alcohol problem; (3) Low risk drinking; and (4) Hazardous drinking. This method (defined in Table 2) is recommended by Babor et al. to obtain a more detailed interpretation of an individual’s total AUDIT score [19]. Percentage in each category of sociodemographic characteristics and AUDIT risk categories were calculated and presented overall, and by site. Logistic regression was used to determine the independent correlates of hazardous drinking. Crude and adjusted odds ratios and 95% confidence intervals were calculated from the logistic regression models. Responses to the eight eSBI feedback questions were summarized using means and standard deviations. Data were analyzed using Stata v15 (StataCorp LLC, College Station, TX).

#### Qualitative Component

FGDs and individual interviews were analyzed separately using English translations of the transcripts. A general inductive approach was used to analyze the qualitative data [24]. Prior to analysis, lists of overarching domains were created separately for FGDs and individual interviews to guide the analyses (e.g. program usability, strengths and weaknesses of the program, program effects, suggestions for program improvements, etc.). AMT and SYH each independently coded one FGD transcript and one interview transcript. An initial coding framework was then developed through discussion. Each transcript was then coded by AMT and independently verified by SYH. When new codes emerged, these were discussed and added to the coding framework. The transcripts were then re-read with the new codes in mind. The final codes were organized into categories and discussed with the team to determine the key themes that emerged.

## Results

### Quantitative Results

A total of 787 participants were recruited, including 390 patients from the Katutura Health Center and 397 patients from Oshakati Hospital. At the baseline visit, 18 patients who began the eSBI program did not complete it (17 from Katutura and 1 from Oshakati), resulting in a total of 769 participants with analyzable data. Sociodemographic and ART characteristics of the participants are presented in Table 1. The average age was 41.2 ± 9.6 with participants in Oshakati being slightly older than participants in Katutura. At both sites, slightly more than half were female and Oshiwambo was the language of choice for the majority. Education and marital status were also similar between sites, with the majority educated at the secondary school level and either married, living with a partner, or in a relationship. Employment status was higher in Katutura (54%) compared to Oshakati (29%). Of those who were employed, the majority received their wages monthly. Household income was lower in Oshakati, with nearly two-thirds living on ≤ 500 Namibian dollars (~ 37 US dollars) per month. Practically all respondents (98%) were on ART in the past month and of those, there was no difference in the total proportion reporting “Excellent” or “Very good” adherence (90% at both sites); however, a higher proportion of participants in Oshakati reported “Excellent” adherence compared to Katutura (70% vs. 59%).

**Table 1:** Sociodemographic and ART characteristics of 769 study participants, overall and by site

	Katutura Health Centre (n = 373)	Oshakati Hospital (n = 396)	Total (n = 769)
Age in years, mean ± SD	40.2 ± 8.1	42.2 ± 10.7	41.2 ± 9.6
Sex			
Female	193 (52%)	254 (64%)	447 (58%)
Primary language			
Afrikaans	23 (6%)	0 (0%)	23 (3%)
English	55 (15%)	5 (1%)	60 (8%)
Oshiwambo	295 (79%)	391 (99%)	686 (89%)
Education			
No school	64 (17%)	50 (13%)	114 (15%)
Primary (grades 1–7)	120 (32%)	129 (33%)	249 (32%)
Secondary (grades 8–10)	132 (35%)	154 (39%)	286 (37%)
Secondary (grades 11–12)	40 (11%)	50 (13%)	90 (12%)
Tertiary or higher	17 (5%)	13 (3%)	30 (4%)
Marital status			
Single	97 (26%)	130 (33%)	227 (30%)
Relationship (not married)	122 (33%)	133 (34%)	255 (33%)
Married or living with partner	138 (37%)	109 (28%)	247 (32%)
Separated or divorced	10 (3%)	9 (2%)	19 (2%)
Widowed	6 (2%)	15 (4%)	21 (3%)
Employment status			
Employed	202 (54%)	116 (29%)	318 (41%)
Wages received^a^			
Daily	8 (4%)	12 (10%)	20 (6%)
Weekly	9 (4%)	4 (3%)	13 (4%)
Monthly	185 (92%)	99 (86%)	284 (90%)
Monthly household income^b^			
0 to 500 NAD	132 (36%)	251 (64%)	383 (50%)
501 to 1000 NAD	102 (27%)	82 (21%)	184 (24%)
1001 to 2500 NAD	86 (23%)	34 (9%)	120 (16%)
2501 to 5000 NAD	37 (10%)	18 (5%)	55 (7%)
> 5000 NAD	14 (4%)	8 (2%)	22 (3%)
On ART (past month)			
No	12 (3%)	6 (2%)	18 (2%)
Yes	361 (97%)	390 (98%)	751 (98%)
Self-reported ART adherence (past month)^c^			
Excellent	213 (59%)	272 (70%)	485 (65%)
Very good	111 (31%)	79 (20%)	190 (25%)
Good	21 (6%)	32 (8%)	53 (7%)
Fair	13 (4%)	5 (1%)	18 (2%)
Poor	3 (1%)	2 (1%)	5 (1%)

^a^Wages received: percentage of employed

^b^Household monthly income: missing data on 2 from Katutura and 3 from Oshakati

^c^ART adherence: percentage of those taking ART

Table 2 shows drinking classifications based on the two different methods outlined in the original WHO AUDIT manual [19]. Using the classification of alcohol risk level based on the AUDIT score, 22% of patients in Katutura and 8% of patients in Oshakati were classified as medium risk or above (AUDIT score ≥ 8). Using the alternative method of classifying respondents according to their answers to specific AUDIT questions, 34% of patients in Katutura and 17% of patients in Oshakati were classified as hazardous drinkers, including 25% and 11% who were alcohol dependent and 24% and 12% experiencing alcohol harm in Katutura and Oshakati, respectively. An additional 8% in Katutura and 13% in Oshakati were non-drinkers but had past or recent (within the past year) alcohol problems. When asked what types of alcohol participants usually consumed, the majority reported drinking beer (53%), followed by “tombo” (30%), a traditional homebrew. Approximately 15% reported drinking wine.

**Table 2:** Drinking classifications according to AUDIT score (A) and specific AUDIT questions (B), overall and by site

Classification	Definition	Katutura (n = 373)	Oshakati (n = 396)	Total (n = 769)
A. Risk level based on AUDIT score				
Low risk	Score: 0–7	293 (79%)	362 (91%)	655 (85%)
Medium risk	Score: 8–15	55 (15%)	25 (6%)	80 (10%)
High risk	Score: 16–19	15 (4%)	5 (1%)	20 (3%)
Dependent	Score: 20–40	10 (3%)	4 (1%)	14 (2%)
B. Risk level based on specific answers to AUDIT questions^a^				
1. Non-drinker, no risk	Not currently drinking; no alcohol related injuries or others concerned about drinking	145 (39%)	201 (51%)	346 (45%)
2. Non-drinker, past or recent alcohol problem	Not currently drinking; past or recent alcohol related injuries or others concerned about drinking	29 (8%)	50 (13%)	79 (10%)
3. Low risk drinking	Currently drinking 1 or 2 drinks typically and never ≥ 6 drinks on one occasion	72 (19%)	76 (19%)	148 (19%)
4. Hazardous drinking	≥ 3 drinks typically or ≥ 6 drinks on at least one occasion	127 (34%)	67 (17%)	194 (25%)
a. Alcohol dependence	Impaired control over drinking; increased salience of drinking; morning drinking	94 (25%)	44 (11%)	138 (18%)
b. Alcohol harm	Guilt after drinking; blackouts; alcohol-related injuries; others concerned about drinking	90 (24%)	48 (12%)	138 (18%)

^a^The first four categories are mutually exclusive (percentages add up to 100%). Alcohol dependence and alcohol harm are subsets of hazardous drinking and are not mutually exclusive

Several factors were associated with hazardous drinking in this population (Table 3). In unadjusted logistic regression models, attending the ART site in Oshakati and being female were both associated with lower odds of hazardous drinking. However, being separated/divorced/widowed, employed, having a higher monthly household income, and reporting less than excellent ART adherence were all significantly associated with a higher odds of hazardous drinking. In the final multivariate logistic regression model, attending the Oshakati ART site and being female remained independently associated with a lower odds of hazardous drinking, while being separated/widowed/divorced, having a monthly household income > $1000 Namibian dollars (NAD), and reporting less than excellent ART adherence remained independently associated with a higher odds of hazardous drinking. There was no significant effect modification by site or gender.

**Table 3:** Correlates of hazardous drinking at baseline

	Hazardous drinking		OR (95% CI)	
	No (n = 575)	Yes (n = 194)	Unadjusted	Adjusted
Age in years (mean ± SD)	41.3 ± 9.8	41.1 ± 8.8	0.99 (0.98, 1.01)	–
Site				
Katutura	246 (66%)	127 (34%)	1.00	1.00
Oshakati	329 (83%)	67 (17%)	0.39 (0.28, 0.55)	0.50 (0.35, 0.72)
Sex				
Male	209 (65%)	113 (35%)	1.00	1.00
Female	366 (82%)	81 (18%)	0.41 (0.29, 0.57)	0.48 (0.34, 0.69)
Education				
No school	88 (77%)	26 (23%)	1.00	–
Primary (grades 1–7)	184 (74%)	65 (26%)	1.20 (0.71, 2.01)	–
Secondary (grades 8–12)	281 (75%)	95 (25%)	1.14 (0.70, 1.88)	–
Tertiary or higher	22 (73%)	8 (27%)	1.23 (0.49, 3.09)	–
Marital status				
Single	179 (79%)	48 (21%)	1.00	1.00
Married/in a relationship	371 (74%)	131 (26%)	1.32 (0.90, 1.92)	1.04 (0.70, 1.56)
Separated/divorced/widowed	25 (63%)	15 (38%)	2.24 (1.09, 4.57)	2.27 (1.04, 4.96)
Employment status				
Unemployed	361 (80%)	90 (20%)	1.00	–
Employed	214 (67%)	104 (33%)	1.94 (1.40, 2.71)	–
Monthly household income^a^				
0 to 500 NAD^b^	314 (82%)	69 (18%)	1.00	1.00
501 to 1000 NAD	133 (72%)	51 (28%)	1.75 (1.15, 2.64)	1.38 (0.89, 2.15)
> 1000 NAD	123 (62%)	74 (38%)	2.74 (1.86, 4.04)	1.86 (1.21, 2.85)
Past month ART adherence^c^				
Excellent	385 (79%)	100 (21%)	1.00	1.00
Less than excellent	179 (67%)	87 (33%)	1.87 (1.34, 2.62)	1.70 (1.19, 2.43)

^a^5 participants responded “I don’t know” or “I don’t want to answer”

^b^NAD = Namibian dollars

^c^18 participants were not taking ART in past month

### Timing of eSBI and Feedback Questions

The average (mean ± SD) amount of time it took participants to complete the eSBI program was 16.5 ± 8.1 min (median = 14.8 min, IQR: 11.3–19.4 min). Hazardous drinkers took 23.1 ± 10.1 min to complete the eSBI compared to 14.3 ± 5.9 min for non-hazardous drinkers. Participant feedback on the usability and acceptability of the eSBI are reported in Table 4. On a Likert scale ranging from 1 (Strongly Agree) to 5 (Strongly Disagree), the means for the feedback questions ranged from 1.27 to 2.04, all of which were in the “Strongly Agree” to “Agree” range (between 1 and 2), indicating highly favorable responses. For all but one question (#3), ≥ 92% of participants endorsed “Strongly Agree” or “Agree”. For question #3, 72% agreed the program was too long, 22% disagreed, and 6% remained neutral.

**Table 4:** Participant feedback on the usability and acceptability of the eSBI program (n = 769)

Feedback statement	Mean (SD)
1. “I thought the computer program was easy to use”	1.27 (0.70)
2. “I felt comfortable using this computer program”	1.34 (0.82)
3. “The computer program was too long”	2.04 (1.40)
4. “I found this computer program helpful”	1.28 (0.69)
5. “The information I received will help me limit my alcohol use”	1.33 (0.83)
6. “I would use this computer program again”	1.36 (0.81)
7. “I would rather use this computer program than speak to the doctor or health worker about my drinking”	1.41 (0.91)
8. “I answered the questions more honestly than I would have if I had been speaking with a doctor or a health worker”	1.32 (0.75)

Responses are based on Likert scale ranging from 1 = strongly agree to 5 = strongly disagree

### Qualitative Results

#### Interviews with Study Participants

The demographic characteristics of the 20 participants interviewed were similar to that of the overall sample with 58% female, mean age of 41.8 ± 10.5 years, the majority speaking Oshiwambo as their primary language, and nearly 90% reporting “Excellent” or “Very Good” ART adherence at baseline. Alcohol intake patterns at baseline were also similar to the overall sample with 21% hazardous drinkers and 47% non-drinkers with no risk. However, more interview participants were non-drinkers with a past/recent alcohol problem (21% vs. 10%) and fewer were low risk drinkers (11% vs. 19%) compared to the overall sample. Approximately 85% of interview participants had two or three follow-up visits where they completed the eSBI program.

##### Program Usability

With respect to the usability of the program, participants were generally very favorable about the program, confirming the quantitative feedback shown in Table 4. Although a few found parts of the program to be difficult,

“It was difficult in the beginning but the interviewer helped me…the computer sometimes I press but nothing changes”—Katutura, female, 45 years old

“The second part was difficult because the questions were being repeated a lot”—Oshakati, female, 44 years old

most found the eSBI easy to navigate and liked the questions, advice, and pictures:

“It was easy to learn”—Katutura, male, 27 years old

“The one speaking in the computer explained it well. She made it very easy to use”—Katutura, female, 42 years old

“I understood the questions and teachings. The explanations were good”—Oshakati, male, 54 years old

“The pictures were very clear and show reality”—Katutura, female, 45 years old

“Yes, it was a great joy for me to watch the pictures in the video. I learned a lot”—Oshakati, female, 40 years old

A substantial minority of participants interviewed felt that the eSBI program was too long.

##### Progam Effects

In describing how the program affected them, many patients reported learning about the hazardous effects of alcohol from the eSBI program, particularly on ART adherence.

*“*It showed me that if I keep drinking, my medicine will not work”—Katutura, male, 46 years old

“It has taught me about the dangers of consuming [alcohol] whilst on ARV treatment”—Katutura, female, 45 years old

“It touched me a lot. When a person is drinking alcohol you always forget to drink your medicine”—Oshakati, female, 40 years old

Some participants indicated following the advice given to them by the eSBI program:

“I used the advice, mostly because it [drinking] could lead to drug resistance and treatment will fail”.—Katutura, male, 27 years old

Examples of specific advice used by participants included:

“I stopped staying with friends who drink”—Oshakati, female, 40 years old

“I left all my bad friends, yes I learned a lot from the advice I received from the computer”—Oshakati, female, 36 years old

“I keep myself busy with other things, because I want to take better care of myself. I had to reduce [my alcohol intake]”—Katutura, female, 45 years old

Among those that reported alcohol use, many reported changing their alcohol behavior since participating in the eSBI program.

“It changed me because I don’t think of alcohol any more…and it’s all because of the program”—Katutura, male, 27 years old

“Yes, alcohol touched my life, but now that I changed I see a difference in my life”—Katutura, female, 36 years old

“I stopped drinking. My life changed and I am grateful”—Oshakati, female, 23 years old

##### Progam Acceptability

Keeping in mind that the majority of patients we interviewed completed the eSBI program at least three times, most did not express difficulties participating in the alcohol program at the follow-up visits; however, several patients in Oshakati mentioned being afraid to participate the first time suggesting that patients may need more information about the program prior to introduction into clinics.

“The first time was very difficult because I was afraid”—Oshakati, female, 48 years old

“It was difficult, I was afraid of the questions. I became happy because of the advice it added to what I know already”—Oshakati, female, 40 years old

We only received a few suggestions from patients on how to improve the eSBI program, including making it shorter, providing it to all patients at the ART sites, and using tablets instead of laptop computers.

#### FGD’s with Providers

The FGD’s with clinic staff (including doctors, nurses, and pharmacists) revealed useful insights on implementation of the eSBI program, including perceived benefits to patients and providers; problems with the eSBI program; and potential improvements for future implementation in ART clinics.

##### Perceived Benefits of the eSBI Program

Providers in Oshakati were able to identify some benefits of the eSBI program to patients; however providers in Katutura did not have direct interaction with patients about the eSBI program so were not able to talk in depth about perceived benefits. In Oshakati, providers perceived that patients were happy with the program because they were speaking to other patients about the program.

“The patients who were interviewed I think they gave feedback to others who were not randomly selected…those not selected would come contacting us to ask when they would be interviewed. I think it was a positive program because if someone is engaged in something and they give positive feedback, then others like the idea of the program and even decided or came to the level of contacting the health workers to ask when am I going to be interviewed, and you have to explain that it’s not done to everyone.”—Oshakati, P5

“The positive was people asking to also get interviewed on alcohol because maybe there are some good things I can learn from it.”—Oshakati, P3

Also in Oshakati, providers felt that the eSBI program was beneficial to them as providers because it was adding to the alcohol counseling they were already providing to the patients and would help them to identify patients for increased monitoring and TB treatment.

“We are already screening our patients when they come for consultation, and during the ongoing morning session, alcohol is one of the topics being touched, so I think adding this program… will help reduce the alcohol consumption and that will lead patients to be able to adhere and they will take their medication correctly at the right time.”—Oshakati, P5

“If you are screening a patient and you are getting the results, then maybe you will see… this person is at high risk, so it will be very beneficial not only to them but also for us to identify those who need additional monitoring”—Oshakati, P1

“It will be beneficial, especially when we are giving the isoniazid and preventative therapy. We don’t give if someone is abusing alcohol and the person will be at risk of getting TB. So if this program is effective then we will give that service to more patients, to prevent TB.”—Oshakati, P4

##### Problems with the eSBI Program

Providers in Katutura identified the lack of contact with participants after the eSBI program as a problem.

“Like I said, we didn’t get to see the results so we can’t tell if it worked or not. When we talk to them about alcohol in the screening rooms, they know we are against alcohol consumption with treatment, so they will never tell us the truth. But we don’t know the reality, because we didn’t see the [eSBI] reports.”—Katutura, P1

Also in Katutura, one provider believed that the reason why patients did not attend eSBI follow-up visits was because it took too much time or because the information was repetitive.

“From what I saw, patients would come in, and they would not come for follow up, they would disappear…because what happens is, we identify them, then we refer them to [the study coordinator], she would get their files and try to get their medication from the pharmacy, but I think the time spent on the study is what makes them run away…and the other thing is it’s almost like a repetition of the same questions. I don’t know, maybe it’s because I went through it [gave patients information on alcohol] so maybe they feel it’s just the same thing…When we are referring them, working at the front table taking their blood pressure and weight, we recognize the eSBI sticker [indicates they are a study participant] and you refer them to that room, they will just say “no, it’s too much” or “I’m in a hurry” they will just say something.”—Katutura, P1

In Oshakati, where providers had access to patients’ eSBI reports, they felt that patients were hesitant to participate in the study because they feared being punished for drinking alcohol.

“There are some who said ‘I don’t want to take part in that study, because otherwise if they learn that I’m consuming alcohol, maybe I will be punished or one day be part of something I don’t want to be part of’”—Oshakati, P3

##### Suggested Improvements for Future Implementation

Several improvements were suggested by providers in both clinics. The first was the need for more staffing support and clinic space.

“The other challenge I think is, the kind of setting we have in this clinic, we had to accommodate two studies in one room, we then had to move [the study] out of that room to create more space for the other clinic functions. The whole thing is ok, its fine with us, but …we will need a lot of support in terms of staffing, we don’t have space any more unless they bring us a [shipping] container.”—Katutura, P1

“It can [be incorporated into routine clinical care], provided that we will add on the staff so that maybe one or two people are responsible for it…we should add people who are screening the people electronically, then have people to counsel them…”—Oshakati, P1

**“**I have the same idea, that we just need extra hands to do that, but it [the eSBI program] is really really needed.”—Oshakati, P5

In addition, the physical placement of the eSBI program at the ART site was thought to be important for maximizing participation. In Oshakati, participants were taken to another building to complete the eSBI.

“It will need to be next to the CDC clinic department, because here it is a bit far. If you refer someone here it will be out of their way. They understand they are going to be joining and I’m going to be there [in another building], they will disappear, more especially those ones who are taking alcohol they are more likely to be stubborn not to show up.”—Oshakati, P1

Providers in Oshakati also suggested that the program be offered late in the afternoon (our study stopped seeing participants at 3 pm each day) to capture the heavy drinkers who tend to come to the clinic late in the afternoon.

“What we noticed at the CDC clinic is that some patients who really consume alcohol come late afternoon, like to 5. Some you can even see I can’t attend to this patient, if I’m talking to him he won’t understand me, so it’s better to tell him to come back tomorrow when he is sober.”—Oshakati, P5

“In support with her point that, many a time, those that really consume alcohol come in the later hours, they are the trouble makers. It could be the reason why the answers or response of the study is coming out as such, the majority seen in the morning are the normal people, they come, wait until 10, go back to work they are the responsible ones. The majority who were supposed to be captured (screened and enrolled), they are not captured because it was not incorporated into their time frame.”—Oshakati, P1

Another suggestion that providers in Oshakati had for improving participation in the program was to provide more education to patients before introducing the program in the clinic.

“One more thing I think needs to improve is maybe to give health education or health talks, before the actual interview takes place, just to educate them on the benefit of the program so that they can understand.”—Oshakati, P3

“I would like to say that you must educate the person first that there is a certain study with a program that will come about a b c d, and this is how you will benefit and this is how you are expected to contribute”—Oshakati, P5

Some final suggestions by providers were to make the program shorter and to offer it less frequently.

“If it can be made a bit shorter, and how often, when it was being designed how often was it meant to be done? Once a year for every patient? Or was it at every visit?”—Katutura, P1

“I also want to add on that we should have a schedule for how long [often] we screen our people for alcohol, because we cannot be doing it on a monthly basis. Like the person came yesterday and a month later you have again screened, so if we could incorporate it like ok, we will be screening our patients after each and every 6 months or 3 months, then I understand there we could really see the impact for it to our patients.”—Oshakati, P1

Finally, based on comments from providers at both sites, we felt that a potential improvement for increasing truthful reporting would be to not give providers access to patients’ eSBI reports. In Katutura, providers were not given access to patients’ reports and one provider felt strongly that if this were to change, patients would not report the truth about their drinking levels:

“It’s a good tool, to have at a clinic, however once the patients know we have access to their reports, the stories will change, so next visit they will start changing them…What they know is that if they are taking alcohol and they miss their follow up dates or there are any adherence issues with treatment, we usually stop their treatment. So most of the time they will try to change the kind of responses they give when you ask them anything to do with alcohol. So if they discover we have access to reports, it will be a totally different thing.”—Katutura, P1

In support of this comment, in Oshakati, where providers *were* given access to patients’ reports, they felt this inhibited patients from telling the truth.

“No, they were not telling the truth [on the computer], like how many beers do you take? Instead of saying five, they will say just a glass. They were not told what is the real aim for them to give the real answer. [When I asked] Why did you not tell them? [Patient responded] I thought maybe I’m going to be punished” [Oshakati, P3]

## Discussion

The results of this study suggest that implementation of alcohol eSBI programs in ART clinics in Namibia can detect hazardous drinking and is potentially feasible. Our low follow-up rates suggest that the intervention was not acceptable in the form that it was implemented. We believe that implementation (and thereby acceptability) could be improved with some programmatic considerations that were revealed in the evaluation phase and described below. Overall, 45% of participants reported some alcohol use in the past 12 months and 25% reported hazardous drinking levels (≥ 3 drinks typically or ≥ 6 drinks on at least one occasion). Eighteen percent were classified as harmful drinkers and 18% as alcohol dependent. Rates of excessive drinking (hazardous, harmful, and/or dependent) were two or more times higher in Katutura than in Oshakati. Hazardous drinkers were also significantly more likely to be male, separated/widowed/divorced, have a monthly household income > $1000 NAD, and to report less than excellent ART adherence.

As part of a PEPFAR-funded initiative to reduce the harmful use of alcohol and mitigate its effects on HIV transmission in sub-Saharan Africa, a series of studies and programmatic activities were conducted in Namibia between 2008 and 2013. Seth et al. conducted a population-based cross-sectional survey of adults living in Katutura and found that 73% reported alcohol consumption in the previous 12 months [25]. Nearly 40% of those surveyed were classified as harmful, hazardous or likely dependent drinkers (AUDIT ≥ 8), including 57% of men and 31% of women. Among current drinkers, 53% of men and 33% of women reported binge drinking on a monthly, weekly or daily basis. In another study, 501 high-risk HIV-negative men seeking services at a voluntary HIV counseling and testing site in Katutura were screened and the average AUDIT score was reported to be in the harmful drinking range (mean = 12.4; 95% CI 12.2–12.7) [26]. A third study among 1,186 PLHIV enrolled across six ART sites in Namibia identified 7.6% with AUDIT scores ≥ 8 [11]. In all three of these studies the AUDIT questionnaire was interviewer-administered. The lower rate of excessive drinking found among PLHIV at ART sites, compared to the general population or to men at high-risk of HIV infection, could be partly due to real reductions in alcohol consumption following HIV diagnosis and ART counseling, and partly due to the fear of disclosing excessive alcohol use in a clinical care setting. In our study, using the same AUDIT questionnaire but administered using an electronic tool without interviewers present, we identified a higher proportion of at-risk drinkers (15%) with AUDIT scores ≥ 8 across two ART sites in Namibia. An alternative method of classifying drinking levels based on answers to specific AUDIT questions yielded an even higher proportion (25%) of hazardous drinkers and provided us with more detailed information on past alcohol problems, alcohol dependence, and alcohol harm. The difference between the two methods of classification indicates that the recommended AUDIT score cutoff of ≥ 8 for risk levels requiring intervention (a cutoff that is widely used by others) may not be appropriate for use in PLHIV in Namibia and requires further validation. In our study, the cutoff score of ≥ 8 resulted in low sensitivity but high specificity. Of the 194 participants categorized as hazardous drinkers (defined as having ≥ 3 drinks typically or ≥ 6 drinks on at least one occasion), 84 (43%) had AUDIT scores < 8. And, of the 575 who were not classified as hazardous drinkers, 571 (99%) had AUDIT scores < 8. A lower AUDIT score cutoff for at-risk drinking (e.g. ≥ 5) would be more sensitive (77%) but still highly specific (95%) when compared to hazardous drinking. Future studies to validate the cutoff scores for at-risk drinking should be considered when using the AUDIT screener in Namibia.

At-risk drinking rates were higher in Katutura than in Oshakati, regardless of which classification method was used. After analyzing the post-intervention FGDs with providers, we hypothesized several reasons that could explain this finding. The first is that patients in Oshakati were still hesitant to report their true levels of alcohol consumption because they knew that the providers would ask for their eSBI reports afterwards. In Katutura, where the response rate for hazardous drinking was higher, we learned that providers did not ask patients for their reports. Providers at both sites felt strongly that once patients think that providers will see their reports, they will not admit to drinking. Another possible reason for the lower prevalence of hazardous drinking in Oshakati was the fact that study recruitment took place mostly in the mornings and early afternoons. Providers at Oshakati pointed out that the patients with more alcohol problems tended to come to the clinic in the late afternoon hours and therefore were less likely to be recruited. A third reason suggested by the providers is that patients in the North (Oshakati) may be distrustful of computers because they are not as familiar with technology as those in Katutura.

Several factors were independently associated with hazardous drinking in our study, including attending the Katutura Health Centre; being male; being separated, widowed, or divorced; having a higher monthly household income; and reporting less than excellent adherence. It is not surprising that men were more likely to report hazardous alcohol use in our study given that alcohol consumption in the general Namibian population is higher among men than women [4, 25]. Excessive alcohol use has also been linked to social factors such as marital/relationship status and income levels in previous studies. Consistent with our findings, many studies have found that men and women who are separated, divorced, or widowed are more likely to report excessive alcohol use. This has been demonstrated in HIV-infected populations [27, 28] and in various other populations in sub-Saharan Africa [29–33]. The evidence is less clear on the link between excessive alcohol use and income level. A systematic review found mixed results on the association between alcohol use and community income and employment levels, but concluded there was some indication that higher levels of alcohol consumption were more likely among adults living in communities with higher income levels [34]. In a study conducted in Tanzania, alcohol use was similarly associated with male gender, being in a relationship, and greater disposable income, among other factors [33]. The association between higher household income levels and hazardous drinking in our study could indicate higher alcohol purchasing power, but this needs further study. The cutoff for the highest monthly household income level was $1000 NAD, equivalent to $81 USD. Only 10% of the study population had monthly income levels > 2500 NAD ($203 USD).

Our eSBI included one question on self-reported ART adherence which was based on a qualitative rating of how well participants felt they were taking their ARVs in the past month. Hazardous alcohol consumption was significantly associated with less than excellent adherence in our study population, a finding that is consistent with many previous studies reporting strong and consistently negative associations between alcohol consumption and ART adherence [12, 35].

Our study had several limitations. First, both the quantitative and qualitative data from patients were derived from convenience samples at two ART sites in Namibia which were purposively selected by the MoHSS; therefore, our results may not be generalizable to HIV patients across the country or even to all patients at the two ART sites. Second, we were only able to interview two of the providers at the ART site in Katutura so we did not get a broad perspective on eSBI implementation at that site. Third, since we did not keep track of precisely how many patients were offered but refused to use the eSBI (although study coordinators reported very few refusals at baseline), we cannot rule out the potential for heavier drinkers to opt out of participating in the study resulting in an underestimate of hazardous drinking. Fourth, since evaluations were conducted at the end of the study period, selection of participants for individual interviews included a large proportion of those who remained in the study; therefore, feedback from these participants may be more positive than those who did not complete the study; however, quantitative feedback from the entire sample was overwhelmingly positive as well. Fifth, follow-up rates in our study were low (see “Programmatic Considerations” section for further discussion) and therefore we were unable to assess changes in alcohol consumption over time. Sixth, we used a single-item Likert-rating scale with 1 month recall to measure ART adherence. While this type of single-item adherence question has low participant burden and has been shown to perform well against more objective measures of adherence [36], it has not been validated in Namibia. Finally, it is difficult to assess whether patients felt comfortable revealing the truth about their alcohol consumption or about the eSBI program. Although our results may underestimate the amount of drinking, we were able to detect a higher proportion of hazardous drinking than previous studies using the same instrument but interviewer-administered.

## Programmatic Considerations

Based on the feedback questions administered at the end of the eSBI program, the vast majority of participants reported that they were able to easily navigate the program, found it helpful, and that the information they received helped them limit their alcohol use. A large majority also agreed they would rather use the computer program than speak to the provider about their drinking, and said they were more honest on the computer. In contrast to these favorable ratings, the high attrition rate was a major concern with regards to feasibility and acceptability of the eSBI in this population. We believe that some of this can be explained by the fact that the follow-up visits straddled the months of December and January, when many Namibians are moving around for family and farming obligations. During the festive season, many HIV patients miss their routine visits at their home clinics, opting for pill pick-up at alternate clinics whilst traveling. However, the in-depth interviews and FGD’s also suggested that many patients were reluctant to repeat the eSBI at their monthly follow-up visits because the study process was too long, the questions repetitive, and the location was inconvenient. The eSBI itself did not take long to complete—half the participants completed it in less than 15 min and three-quarters completed it in less than 19 min. However, in the context of a study, participants also had to go through the screening and informed consent process in a separate room which likely added a substantial amount of time to the entire procedure. Future implementation in the context of a program could alleviate some of these problems by streamlining the process, having more computer tablets available in more convenient locations in the clinic, and allowing patients to initiate eSBI screening anytime during their clinic visit.

Another consideration is the need for more patient education around the introduction of the eSBI in the clinic to encourage truthfulness in reporting. Providers felt that patients did not fully understand the benefits of the eSBI program for helping to reduce excessive drinking and therefore were afraid to reveal their true drinking levels. Therefore, prior to introducing the eSBI into any ART clinic, a peer education campaign with patient advocates should be considered. Furthermore, the providers felt that having access to patients’ reports hindered the ability of patients to tell the truth. Future implementation of eSBI in ART clinics should consider not giving providers universal access to patients’ reports, as previous studies have shown that self-report of alcohol use is reliable when confidentiality and anonymity are assured [37]. Patients should be allowed to choose whether or not to share their reports with their providers and the eSBI program can refer them for additional help, if desired. Appointing an on-site social worker with expertise in alcohol counseling and independent of ART providers should be considered.

Another important programmatic consideration is the frequency at which to administer the eSBI. Monthly administration may be too frequent as evidenced in our study. Less frequent administration of the eSBI program (perhaps every 3 or 6 months) may be adequate, may reduce the tediousness of the program, and may increase uptake at follow-up visits. Finally, having access to more eSBI computers at each site will improve patient flow and prevent bottle-necks and extended wait times.

In conclusion, HIV patients using the eSBI for the first time found it to be a positive and beneficial experience. Through this pilot project, we were able to identify a substantial number of hazardous drinkers and learned some important lessons on program implementation. A future clinical trial to determine the effectiveness of the eSBI at reducing hazardous alcohol consumption in patients on ART is warranted.
